# Fractures of the Femur

**Published:** 1918-09

**Authors:** 


### FRACTURES OF THE FEMUR.

The first session was devoted to a symposium on the "Treatment of Fractures of the Femur", which was participated in by Médecin-Major Leriche and Médecin-Major Heitz-Boyer of the French Army, Major Sinclair of the British Army and Lt.-Col. Blake of the American Expeditionary Forces. It was brought out in the discussion that one of the causes of the bad results that have occurred in treating fractures of the femur has been that the medical officers who first applied the splints had not been properly trained in using them, or else were careless in applying them. One of the speakers said that in view of the fact that fractures of the femur are so frequent in modern warfare and that the result of treatment depends so much upon the first dressing, which might have to be done by any medical officer at the Front, it should be the rule of the army not to promote any medical officer who could not apply a Thomas splint properly. It was further suggested that between battles medical officers should practice applying splints in order to be proficient in times of emergency.

It was also brought out in the discussion that the Thomas splint was in general use in the British and American armies, and that it was used and thought highly of by the French surgeons, but that other splints for treatment of fractures of the femur were used and considered standard in the French army. The apparatus exhibited by Major Heitz-Boyer showed great ingenuity, and seemed admirably adapted to a special group of cases, but he also spoke favorably of the Thomas splint.

It was evident from the discussion by the representatives of the French, British and American armies that they are not far apart in their methods of treating fractures of the femur. It therefore seems probable that a standard apparatus might be decided upon, which could be used in the three armies. This was thought to be highly desirable by the Research Committee for the following reasons :

1. After a battle, wounded soldiers of the French, British and American armies are often cared for in each other's hospitals and are later transported to the hospitals of the army to which they belong.

2. If one method is employed in the three armies — not necessarily to the exclusion of other methods — the splints and apparatus with which French, British and American medical officers are familiar are always on hand in the supply depots.

3. It is better for medical officers to learn thoroughly one acceptable method of treating fractures of the femur than to attempt to use several different splints and therefore not become proficient in handling any of them.

4. Since American physicians are on duty with all three armies, it is important for them to have the privilege of using one method, because if transferred to another service or if called upon to treat cases of fractures of the femur of soldiers of another Army, they should be familiar with the method followed by its surgeons.

The Research Committee therefore decided to request the Chief of the Service de Santé of the French Army, the Director General of the Medical Services of the British Army and the Chief-Surgeon of the American Forces, each to appoint a representative, thus making a Committee of three to agree upon a standard method of treating fractures of the femur which may be adopted in the three armies. The following officers have been appointed : Lt.-Col. Blake, M. R. C., American Expeditionary Forces, Médecin-Major Heitz-Boyer, French Army, Major Sinclair, B. A. in France. They will probably be able to make a report at the October meeting of the Research Committee.

